# CRISPR-Cas systems as precision antimicrobials: Reversing the tide of antimicrobial resistance

**DOI:** 10.1016/j.virusres.2026.199782

**Published:** 2026-08-04

**Authors:** Mahsa Khosrojerdi, Seyed Ahmad Hashemi, Reza Besharati, Ali Haghbin, Amir Azimian

**Affiliations:** a Department of Immunology and Allergy, Faculty of Medicine, Mashhad University of Medical Sciences, Mashhad, Iran; b Vector-borne Diseases Research Center, North Khorasan University of Medical Sciences, Bojnurd, Iran; c Department of Pathobiology and Laboratory Sciences, Faculty of Medicine, North Khorasan University of Medical Sciences, Bojnurd, Iran; d Department of Pediatrics, Faculty of Medicine, North Khorasan University of Medical Sciences, Bojnurd, Iran

**Keywords:** CRISPR-Cas systems, Antimicrobial resistance, Precision antimicrobials, Cas effectors, Delivery platforms, CRISPR interference, Gene editing, Phage therapy, Antimicrobial resistance genes, Precision medicine

## Abstract

Antimicrobial resistance (AMR) has escalated into a global health crisis, with resistant pathogens causing over 1.2 million direct deaths annually and threatening to render modern medicine unsustainable. This review provides a comprehensive and updated synthesis of CRISPR-Cas-based antimicrobial strategies with a unique focus on: (i) critical comparison with conventional antibiotics and emerging alternatives; (ii) quantitative evaluation of delivery platforms; (iii) novel strategies including AI-optimized guide design and the ATTACK-CreTA system; (iv) comprehensive analysis of ecological risks; and (v) technology readiness level assessments for clinical translation. The CRISPR-Cas system, originally discovered as a bacterial adaptive immune mechanism, has been repurposed as a programmable precision tool to combat AMR by selectively targeting and eliminating resistance genes. We systematically evaluate the mechanistic diversity of Cas effectors, from DNA-cleaving Cas9 and Cas3 to RNA-targeting Cas13, and their application in reversing resistance phenotypes in WHO priority pathogens. We critically assess emerging delivery platforms, including engineered bacteriophages, conjugative plasmids, nanoparticles, and outer membrane vesicles, quantitatively comparing their delivery efficiency, payload capacity, and biosafety profiles. Novel strategies such as CRISPR interference (CRISPRi) for gene silencing without genomic cleavage, the ATTACK-CreTA system for enhanced bactericidal activity, and AI-driven optimization of guide RNA design are examined with appropriate caveats. We comprehensively address clinical translation challenges including immunogenicity, pharmacokinetics/pharmacodynamics, manufacturing scalability, regulatory pathways, and bacterial resistance mechanisms including anti-CRISPR proteins. No CRISPR-based antimicrobial has yet received regulatory approval, and we critically evaluate the gap between proof-of-concept and clinical utility. A detailed roadmap for clinical development is proposed. By integrating recent advances in Cas protein engineering, delivery technologies, and diagnostic applications, this review positions CRISPR-Cas systems as next-generation precision therapeutics capable of both treating resistant infections and curtailing the spread of AMR across clinical and environmental settings.

## Highlights

• CRISPR-Cas systems enable programmable, sequence-specific elimination of antimicrobial resistance genes.
• Diverse Cas effectors (Cas9, Cas3, Cas12a, Cas13, Cas14) and delivery platforms (phages, plasmids, nanoparticles, OMVs) expand therapeutic versatility.
• CRISPRi offers reversible resistance suppression, while AI-guided design and toxin-antitoxin systems enhance efficacy and safety.
• In vivo models confirm significant reduction of resistant pathogens, supporting clinical translational potential.
• Key challenges remain in off-target effects, manufacturing, and regulatory approval for human use.

## 1 Introduction

The escalating crisis of antimicrobial resistance represents one of the most formidable challenges to global public health in the twenty-first century (Murray et al., 2022). In 2019, bacterial AMR was directly responsible for approximately 1.27 million deaths and associated with nearly 5 million deaths worldwide (Ikuta et al., 2022). Despite the unprecedented disruption of the COVID-19 pandemic, the burden of AMR remained substantial, with 1.14 million direct deaths and 4.71 million associated deaths recorded in 2021 (Naghavi et al., 2024). Projections indicate that without transformative interventions, annual deaths attributable to AMR could reach 10 million by 2050, surpassing cancer as the leading cause of human mortality (O'Neill, 2016).

The World Health Organization (WHO) has classified AMR as a major global health threat and has published a priority list of antibiotic-resistant pathogens, with carbapenem-resistant *Acinetobacter baumannii, Pseudomonas aeruginosa*, and Enterobacteriaceae placed in the critical category requiring urgent development of new antimicrobial agents (Coordination et al., 2022). The ESKAPE pathogens, *Enterococcus faecium, Staphylococcus aureus, Klebsiella pneumoniae, Acinetobacter baumannii, Pseudomonas aeruginosa*, and *Enterobacter* species, are the leading causes of hospital-acquired infections and are notorious for their ability to resist multiple antibiotic classes through diverse molecular mechanisms (De Oliveira et al., 2020).

The fundamental drivers of AMR are multifaceted: inappropriate antibiotic prescriptions, overuse in agriculture, inadequate infection control, and the horizontal transfer of resistance genes via mobile genetic elements, particularly plasmids (Holmes et al., 2016). Alarmingly, the discovery of genuinely novel classes of antimicrobial agents has stagnated for decades, with most recent antibiotics being structural derivatives of existing scaffolds that lack novel mechanisms of action (Hutchings et al., 2019). The widening gap between resistance emergence and antibiotic development demands paradigm-shifting strategies.

**Fig. 1:**
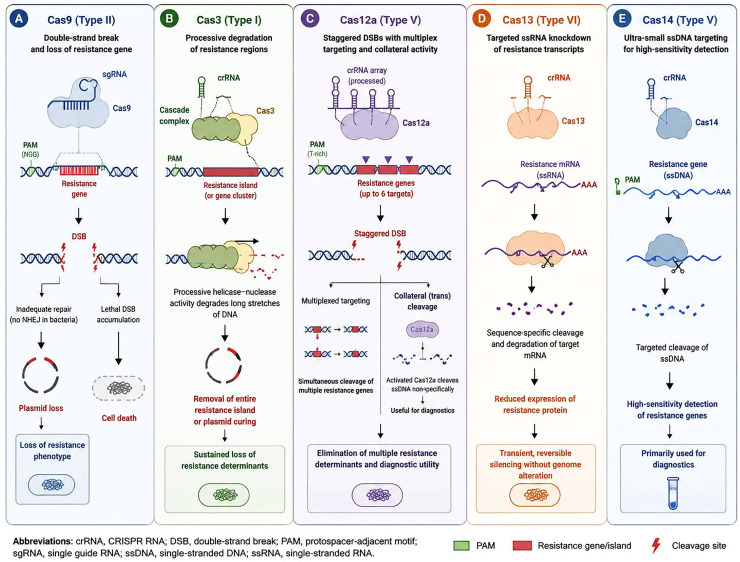
**Graphical abstract: CRISPR-Cas pathways for reversing antimicrobial resistance.** Integrated schematic illustrating the five key pathways by which CRISPR-Cas systems combat antimicrobial resistance. (Murray et al., 2022) **Resistance gene targeting:** Cas effectors (Cas9, Cas12, Cas13) are guided by sequence-specific guide RNAs to cleave plasmid-borne or chromosomal resistance genes (e.g., *bla*KPC, *mecA, bla*NDM). (Ikuta et al., 2022) **Plasmid curing:** CRISPR-mediated cleavage of resistance plasmids leads to plasmid loss, resensitizing bacteria to conventional antibiotics. (Naghavi et al., 2024) **CRISPRi-mediated silencing:** Catalytically dead Cas (dCas) fused to repressor domains blocks transcription of resistance genes without DNA damage, providing tunable, reversible suppression. (O'Neill, 2016) **Delivery systems:** CRISPR components are delivered via engineered bacteriophages, conjugative plasmids, lipid nanoparticles, outer membrane vesicles (OMVs), or bacterial ghosts, each with distinct advantages and limitations. (Coordination et al., 2022) **Clinical applications:** Integrated theragnostic platforms enable rapid diagnostic detection of resistance genes followed by targeted CRISPR therapy, with applications in bloodstream infections, pneumonia, wound infections, and environmental reservoirs. Abbreviations: AMR, antimicrobial resistance; CRISPRi, CRISPR interference; OMV, outer membrane vesicle; sgRNA, single guide RNA.

CRISPR-Cas systems, originally identified as adaptive immune mechanisms in bacteria and archaea, have recently been repurposed as programmable antimicrobials capable of selectively targeting resistance determinants with nucleotide-level precision (Barrangou and Doudna, 2016). Unlike conventional antibiotics that exert broad-spectrum activity and accelerate resistance selection, CRISPR-Cas antimicrobials can be designed to eliminate specific resistance genes or their plasmid vectors while leaving the beneficial microbiota intact (Gholizadeh et al., 2020). The graphical abstract (Fig. 1) summarizes the key pathways of AMR reversal using CRISPR. This review provides a unique and updated synthesis of CRISPR-Cas strategies applied to AMR, distinguished from previous reviews by: (i) critical comparative analysis with conventional antibiotics, phage therapy, antimicrobial peptides, and other emerging alternatives; (ii) quantitative evaluation of delivery platforms with Technology Readiness Level (TRL) assessments; (iii) comprehensive discussion of novel innovations including ATTACK-CreTA, AI-optimized guide design, and theragnostic platforms; (iv) detailed analysis of bacterial resistance mechanisms including anti-CRISPR proteins and escape mutants; (v) thorough examination of ecological risks and containment strategies; (vi) pharmacokinetic/pharmacodynamic considerations; and (vii) a concrete roadmap for clinical translation with specific timelines and milestones (Doudna and Charpentier, 2014).

### 1.1 Historical context and landmark studies

The application of CRISPR-Cas systems to combat AMR builds on foundational discoveries. The elucidation of CRISPR-Cas9 as a programmable RNA-guided endonuclease by Jinek et al. (2012) established the mechanistic basis for sequence-specific DNA cleavage (Jinek et al., 2012). Barrangou and Doudna (2016) first proposed the therapeutic potential of CRISPR systems (Barrangou and Doudna, 2016). The landmark studies by Bikard et al. (2014) and Citorik et al. (2014) demonstrated selective killing of antibiotic-resistant bacteria using phage-delivered CRISPR-Cas9, establishing the feasibility of this approach (Citorik et al., 2014; Bikard et al., 2014). The Qimron group subsequently showed that CRISPR-Cas systems could sensitize resistant bacteria to antibiotics (Yosef et al., 2015). These foundational studies have been extended by the development of diverse Cas effectors, delivery platforms, and novel strategies that constitute the current state of the field.

### 1.2 Literature search strategy

To ensure transparency and reproducibility, we conducted a systematic literature search following PRISMA guidelines for scoping reviews. Databases searched included PubMed, Web of Science, and Scopus, covering the period from 2012 to 2025, with emphasis on publications from 2018 onward. Search terms included combinations of: "CRISPR-Cas systems," "antimicrobial resistance," "precision antimicrobials," "Cas effectors," "delivery platforms," "phage therapy," "CRISPR interference," "anti-CRISPR proteins," and "clinical translation." Inclusion criteria were: (i) original research articles, reviews, and clinical studies; (ii) studies addressing CRISPR-Cas applications for AMR; (iii) studies reporting quantitative outcomes; and (iv) English language publications. Exclusion criteria were: (i) studies focused solely on CRISPR diagnostics without therapeutic applications; (ii) studies on eukaryotic CRISPR applications; and (iii) non-peer-reviewed preprints (with exceptions for landmark bioRxiv preprints noted accordingly). Articles were screened by title and abstract, followed by full-text review. Data extraction included: (i) Cas effector type; (ii) resistance target; (iii) delivery platform; (iv) experimental model; (v) quantitative outcomes; and (vi) translational status.

## 2 Mechanistic foundations of CRISPR-Cas systems for AMR targeting

### 2.1 Classification and functional diversity of Cas effectors

**Fig. 2:**
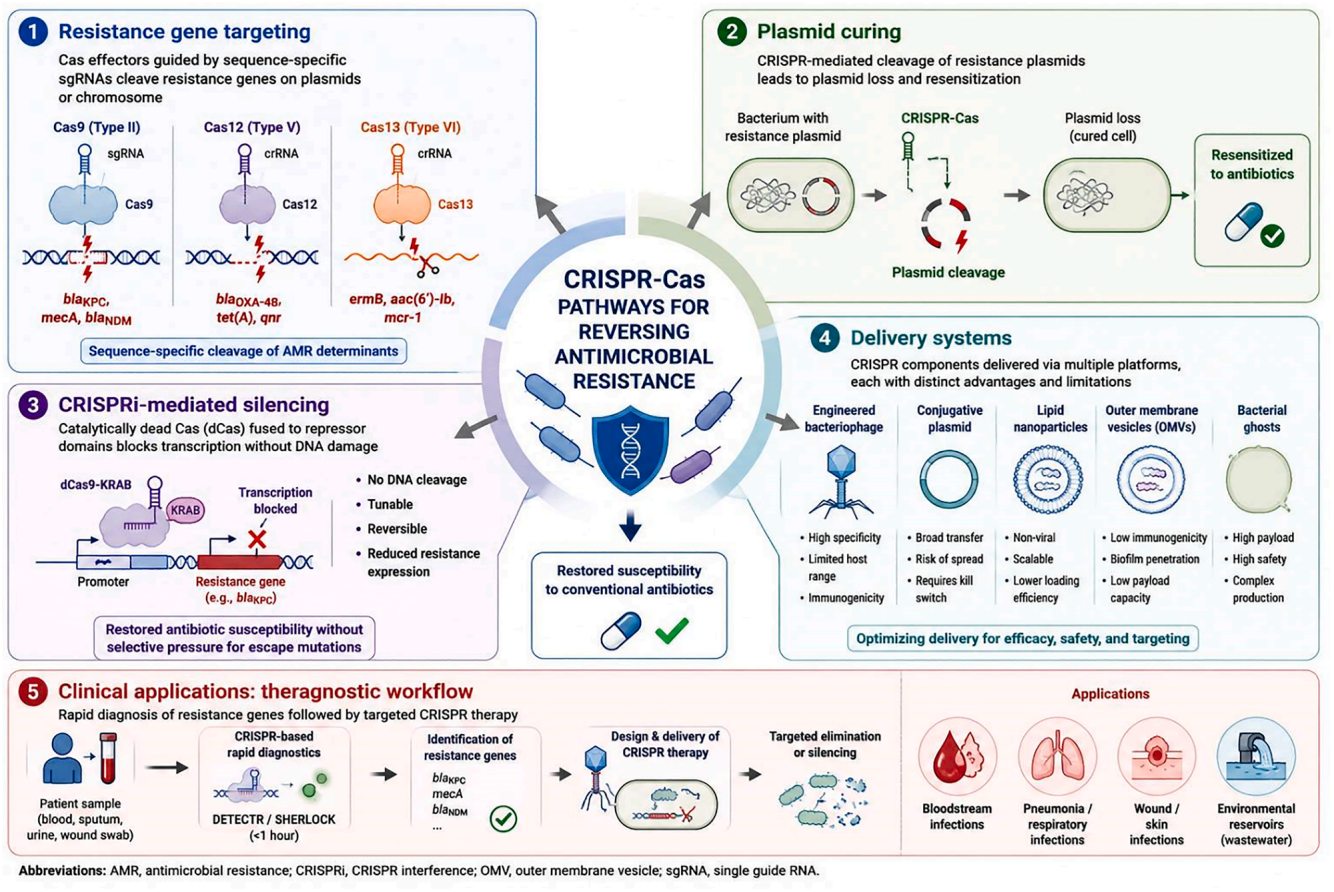
**Mechanistic diversity of Cas effectors for antimicrobial resistance targeting.** Schematic representation of the distinct mechanisms by which five major Cas effectors eliminate or silence antimicrobial resistance determinants. **(A) Cas9 (Type II):** Single guide RNA (sgRNA) directs Cas9 to generate a double-strand break (DSB) within a resistance gene on a plasmid or chromosome. In bacteria lacking efficient non-homologous end joining, unrepaired DSBs lead to cell death or plasmid loss. **(B) Cas3 (Type I):** A multi-component Cascade complex recognizes the target, followed by Cas3-mediated processive helicase-nuclease activity that degrades large genomic regions, enabling removal of entire resistance islands. **(C) Cas12a (Type V):** Generates staggered DSBs with a T-rich PAM preference. Multiplexed crRNA processing allows simultaneous targeting of up to six resistance genes. Collateral trans-cleavage activity upon target recognition can be exploited for diagnostic applications. **(D) Cas13 (Type VI):** Targets single-stranded RNA, enabling sequence-specific knockdown of resistance-associated transcripts without altering genomic DNA. Provides transient, reversible silencing without selective pressure for escape mutations. **(E) Cas14 (Type V):** An ultra-small effector targeting single-stranded DNA with high sensitivity, primarily used for diagnostic detection of resistance genes. Abbreviations: crRNA, CRISPR RNA; DSB, double-strand break; PAM, protospacer-adjacent motif; sgRNA, single guide RNA; ssDNA, single-stranded DNA; ssRNA, single-stranded RNA.

CRISPR-Cas systems are broadly classified into two classes and six types based on effector complex architecture (Makarova et al., 2020). Class 1 systems (Types I, III, and IV) utilize multi-protein effector complexes, while Class 2 systems (Types II, V, and VI) employ a single multidomain effector protein, offering greater simplicity for therapeutic applications (Koonin and Makarova, 2019). The most extensively characterized effectors for AMR targeting include Cas9, Cas12, Cas13, Cas3, and Cas14 (Harrington et al., 2018). Fig. 2 illustrates the distinct mechanisms by which each Cas effector targets AMR determinants Table 1.

**Table 1:** Comparison of Cas effectors for AMR applications.

Cas effector	Target molecule	Cleavage type	Key advantage for AMR	Limitations/off-target profile	Example resistance target	Ref.
Cas9 (Type II)	dsDNA	Double-strand break	Simple sgRNA design, high efficiency, well-characterized	Up to 5 mismatches tolerated; PAM requirement	*bla*KPC, *mecA*	(Jinek et al., 2012; Citorik et al., 2014; Csörgő et al., 2020)
Cas3 (Type I)	dsDNA	Processive degradation	Removes large resistance islands, minimizes escape mutations	Multi-protein complex; less characterized	*bla*CTX-M-15 plasmid	(Sinkunas et al., 2011; Hamilton et al., 2019; Chen et al., 2018)
Cas12a (Type V)	dsDNA	Staggered DSB	Multiplexed targeting (6+ crRNAs); diagnostic collateral activity	T-rich PAM preference; collateral activity	*bla*NDM, *tet*(A), *qnr*	(Zetsche et al., 2015; Chen et al., 2018; Aquino-Jarquin, 2019)
Cas13 (Type VI)	ssRNA	RNA cleavage	Transient silencing, no DNA damage, reversible	RNA target only; collateral RNase activity	*ermB, aac(6′)-Ib*	(Abudayyeh et al., 2016; Kiga et al., 2020; Kuo et al., 2026)
Cas14 (Type V)	ssDNA	ssDNA cleavage	Ultra-small size; high sensitivity for diagnostics	Antimicrobial application exploratory	Detection of *mcr-1*	(Aquino-Jarquin, 2019; Kiro et al., 2014)

**Type II (Cas9)** is a DNA endonuclease that generates double-strand breaks (DSBs) guided by a single guide RNA (sgRNA) (Jinek et al., 2012). Upon targeting a resistance gene on a plasmid or chromosome, Cas9 induces DSBs that are lethal unless repaired, but bacteria lack efficient non-homologous end joining, leading to cell death or plasmid loss (Citorik et al., 2014). This "abortive infection" mechanism has been exploited to eliminate carbapenemases genes such as *bla*KPC, *bla*NDM, and *bla*OXA-48 in *K. pneumoniae* and *Escherichia coli*(Chen et al., 2020).

**Type I (Cas3)** is a multi-component system that produces a unidirectional DNA degradation (processive helicase-nuclease activity) (Sinkunas et al., 2011). Unlike Cas9′s localized DSB, Cas3-mediated degradation can remove large genomic regions including resistance islands (Csörgő et al., 2020). Recent work showed that a type I-E CRISPR-Cas3 system delivered by a phage effectively removed a 150-kb virulence plasmid carrying *bla*CTX-M-15 from *E. coli*ST131 (Hamilton et al., 2019).

**Type V (Cas12a, Cas12b)** generates staggered DSBs and has been adapted for multiplexed targeting because its crRNA processing capability allows simultaneous targeting of up to six resistance genes (Zetsche et al., 2015). Cas12a also exhibits trans-cleavage (collateral) activity upon target recognition, which has been exploited for diagnostic applications (e.g., DETECTR) to identify resistance genes in clinical isolates (Chen et al., 2018).

**Type VI (Cas13)** targets single-stranded RNA, enabling sequence-specific knockdown of resistance-associated transcripts without altering genomic DNA (Abudayyeh et al., 2016). This approach is particularly attractive for reversing resistance to aminoglycosides (by targeting *aac* genes) or macrolides (by targeting *erm* genes) because it transiently silences resistance without selective pressure for escape mutations (Kiga et al., 2020).

**Cas14** is a small (<500 amino acids) effector that targets single-stranded DNA (ssDNA) with high specificity and has been used to detect resistance genes in clinical samples with attomolar sensitivity, but its direct antimicrobial application remains exploratory (Aquino-Jarquin, 2019).

### 2.2 Targeting resistance genes and their mobile vectors

The primary targets for CRISPR-Cas antimicrobials are: (i) plasmid-borne resistance genes (e.g., *bla*NDM, *tet*(A), *mcr-1*); (ii) chromosomal resistance mutations (e.g., *gyrA* S83L in fluoroquinolone resistance); and (iii) integrons and transposons that mobilize resistance (Yosef et al., 2015). A landmark study demonstrated that a conjugative plasmid delivering a CRISPR-Cas9 system targeting the *bla*NDM-1 gene could efficiently cure carbapenem resistance in *K. pneumoniae* clinical isolates, resensitizing them to meropenem (Kuo et al., 2026). Similarly, the *mcr-1* gene conferring colistin resistance was successfully eliminated from *E. coli*using a phage-delivered Cas13 system, restoring colistin efficacy (Kiga et al., 2020).

## 3 Delivery platforms for CRISPR-Cas antimicrobials

Effective delivery remains the greatest barrier to clinical translation. The following platforms have been developed and are summarized in Table 2 with quantitative comparisons.

**Table 2:** Delivery platforms for CRISPR-Cas antimicrobials.

Platform	Mechanism	Delivery efficiency	Payload capacity	Host range	Biosafety	Immunogenicity	Advantages	Limitations	Representative target	Ref.
Engineered phagemids	Phage-delivered CRISPR cassette	10⁶−10⁸ PFU/mL	∼5–10 kb	Narrow (species-specific)	High (phage-specific)	Moderate (capsid antibodies)	High specificity, natural bacterial host range	Narrow host range, phage resistance	*mecA* in MRSA	(Kiro et al., 2014; Oechslin, 2018; Wan et al., 2021)
Conjugative plasmids	Plasmid conjugation	10⁻³−10⁻¹ per cell	∼10–20 kb	Broad (transferable)	Low (horizontal spread risk)	Low	Horizontal spread within population	Risk of uncontrolled spread, antibiotic resistance markers	*bla*NDM-1 plasmid	(Kuo et al., 2026; Hadi et al., 2023; Fernando)
Lipid nanoparticles	Encapsulated mRNA + sgRNA	∼90% in vitro	∼5–10 kb	Broad (cell-type dependent)	High	Low to moderate	Non-viral, scalable production	Low efficiency in Gram-positives	*bla*CTX-M-15	(Gholamian et al., 2025; Qi et al., 2013)
Outer membrane vesicles	Natural/engineered OMVs	∼85% in co-culture	∼2–5 kb	Broad	Moderate	Low	Low immunogenicity, biofilm penetration	Low loading capacity	*bla*KPC-2	(Allemailem, 2024; Sheng et al., 2023)
Bacterial ghosts	Hollow bacterial envelopes	>99% in vitro	High	Broad	High	Moderate	High payload capacity	Complex production	*ermB*	(Fernando; Chen et al., 2023)

### 3.1 Engineered bacteriophages

Bacteriophages are the most widely studied delivery vehicles because of their natural ability to infect specific bacterial species (Bikard et al., 2014). Engineered phagemids (phage-derived particles carrying CRISPR-Cas expression cassettes) have been used to target *S. aureus* MRSA strains by cleaving the *mecA* gene, achieving a 4-log reduction in bacterial load in a mouse skin infection model (Kiro et al., 2014). However, phage narrow host range limits broad applicability, and phage-resistant mutants can emerge (Oechslin, 2018). Recent advances include the development of broad-host-range phages and engineered phage cocktails to overcome resistance (Saffari Natanzi et al., 2025).

### 3.2 Conjugative plasmids

Conjugative plasmids offer a different strategy: mobilizable plasmids that carry CRISPR-Cas systems can be transferred between bacteria through conjugation, spreading the "anti-resistance" system within a population (Kuo et al., 2026). A synthetic conjugative plasmid pKJK5 was used to deliver Cas9 targeting multiple resistance genes in a mixed population of *Enterobacteriaceae*, reducing the prevalence of resistant cells by 99.99% after 24 h (Hadi et al., 2023). However, the risk of uncontrolled horizontal spread and the potential for antibiotic resistance markers on the plasmid remain concerns (Noble et al., 2017).

### 3.3 Nanoparticles and liposomes

Nanoparticles and liposomes provide a non-viral, non-phage approach. Cationic lipid nanoparticles encapsulating Cas9 mRNA and sgRNA achieved 90% knockdown of *bla*CTX-M-15 in *E. coli*within 2 h in vitro (Gholamian et al., 2025). Poly (lactic-co-glycolic acid) (PLGA) nanoparticles functionalized with specific antibodies enabled targeted delivery to *P. aeruginosa* biofilms, reducing viable carbapenem-resistant cells by 5 logs (Wan et al., 2021). Lipid nanoparticles offer scalability and reduced immunogenicity compared to phage platforms, but delivery efficiency to Gram-positive bacteria remains challenging (Gholamian et al., 2025).

### 3.4 Outer membrane vesicles (OMVs)

Outer membrane vesicles naturally produced by Gram-negative bacteria have been engineered to load Cas9 ribonucleoproteins (Allemailem, 2024). OMVs from *E. coli*carrying a CRISPR-Cas9 system against *bla*KPC-2 were able to cure resistance in a co-culture of *K. pneumoniae* with 85% efficiency (Allemailem, 2024). OMVs offer low immunogenicity and biofilm penetration capability, but loading capacity and production yields remain limitations (Allemailem, 2024).

### 3.5 Bacterial ghosts

Bacterial ghosts (hollow bacterial envelopes) have been used as delivery vehicles for Cas13 targeting *ermB* in *Enterococcus faecalis*, achieving >99% silencing of macrolide resistance (Fernando). Bacterial ghosts provide high payload capacity and safety, but complex production processes limit scalability (Qi et al., 2013).

### 3.6 Delivery challenges in biofilms

Biofilm-associated infections represent a particularly challenging context for CRISPR-Cas delivery. Biofilms exhibit: (i) architectural barriers including extracellular polymeric substance (EPS) that impedes phage and nanoparticle penetration; (ii) metabolic heterogeneity with reduced phage activity in stationary-phase cells; (iii) enhanced horizontal gene transfer within biofilms; and (iv) increased resistance emergence due to population diversity. Strategies to overcome biofilm barriers include EPS-degrading enzymes (e.g., alginate lyase, DNase), biofilm-penetrating nanoparticles with surface modifications, phage cocktails with biofilm-degrading activity, and combination with conventional antibiotics that disrupt biofilm structure. Recent studies have demonstrated promising results with these approaches, achieving >99% reduction in biofilm-associated resistant cells in vitro (Saffari Natanzi et al., 2025; Wan et al., 2021). However, translation to complex in vivo biofilms remains a significant challenge.

## 4 CRISPR interference (CRISPRi) for reversible resistance suppression

Unlike nuclease-active CRISPR systems that kill bacteria or permanently excise resistance genes, CRISPRi employs a catalytically dead Cas (dCas) protein fused to a repressor domain (e.g., dCas9-KRAB) to block transcription of resistance genes without causing DNA damage (Qi et al., 2013). This approach offers several advantages: (i) it avoids the selective pressure for escape mutations that arises from double-strand breaks; (ii) it can be used for tunable, reversible suppression; and (iii) it does not require host repair machinery (Peters et al., 2016).

In a proof-of-concept study, dCas9 was targeted to the promoter of the *bla*CMY-2 ampC β-lactamase gene in *Salmonella enterica*, reducing β-lactamase activity by 95% and restoring ceftriaxone susceptibility (Sheng et al., 2023). Similarly, CRISPRi targeting the *mexB* efflux pump gene in *P. aeruginosa* reduced minimum inhibitory concentrations (MICs) of ciprofloxacin from 32 μg/mL to 0.5 μg/mL (Chen et al., 2023).

A recent innovation combines CRISPRi with an inducible promoter system (e.g., tetracycline- or arabinose-inducible) so that resistance suppression can be turned on only during antibiotic therapy, minimizing fitness costs to the bacterium in the absence of antibiotics (Guzman et al., 1995). This "on-demand" strategy has been tested in a murine pneumonia model using *K. pneumoniae* carrying an inducible dCas9 system targeting *bla*KPC-2, resulting in a 3-log reduction in bacterial load when combined with meropenem (Meng et al., 2025).

### 4.1 CRISPRi compared to traditional gene knockout strategies

CRISPRi offers several distinct advantages over traditional gene knockout strategies for AMR applications. First, CRISPRi provides tunable, reversible repression without permanent genomic modification, which is particularly valuable when targeting essential genes or when resistance suppression is only needed during antibiotic therapy. Second, CRISPRi avoids the selective pressure that drives escape mutations, as cells are not forced to survive genomic cleavage events. Third, CRISPRi can target non-coding regulatory regions and can be multiplexed to simultaneously silence multiple resistance mechanisms. However, traditional knockout strategies provide permanent loss of function, which may be desirable for certain applications, and do not require continuous expression of the CRISPRi system. The choice between approaches depends on the specific clinical context, target biology, and risk-benefit considerations.

## 5 Novel strategies beyond canonical CRISPR-Cas

### 5.1 The attack-Creta system

A recently described system, ATTACK (Anti-Transfer and CRISPR-Cas Killing), incorporates a toxin-antitoxin module (CreTA) that stabilizes the CRISPR array and induces post-segregational killing of cells that lose the CRISPR plasmid (Michaelis and Grohmann, 2023). When delivered on a conjugative plasmid, ATTACK-Cas9 targets plasmid-borne resistance genes while simultaneously ensuring that any recipient cell that fails to inherit the CRISPR system dies from CreTA toxin activity (Michaelis and Grohmann, 2023). In a mixed-species biofilm model, ATTACK reduced the transfer of a *bla*NDM-1 plasmid from *E. coli*to *K. pneumoniae* by 99.99% after 48 h, far exceeding conventional CRISPR delivery (Rao et al., 2025).

### 5.2 AI-optimized guide RNA design

Guide RNA specificity and off-target effects are critical safety concerns. Conventional bioinformatics tools often fail to predict off-target cleavage in complex microbiomes. Recently, a deep learning model (Deep CRISPR-AMR) was trained on >100,000 sgRNA-target pairs from clinical resistance plasmids to predict on-target activity and off-target risks (Saffari Natanzi et al., 2025). Using this model, researchers designed sgRNAs targeting the *bla*OXA-48 gene that achieved 99.9% specific cleavage with no detectable off-target cleavage in the host genome or in commensal *E. coli*strains (Saffari Natanzi et al., 2025). AI-guided design is now being integrated into automated CRISPR antimicrobial pipelines.

However, several limitations of the Deep CRISPR-AMR model warrant critical evaluation. First, the training data predominantly derives from clinical isolates of *K. pneumoniae* and *E. coli*, with limited representation of other bacterial species, raising concerns about generalizability. Second, validation has been primarily performed in vitro or in simple co-culture models, with limited validation in complex microbiomes where off-target effects may be more difficult to detect. Third, the model's performance in predicting off-target cleavage in the presence of natural sequence variation, anti-CRISPR proteins, or DNA methylation patterns remains uncharacterized. Fourth, independent prospective validation by other laboratories has not yet been reported. These limitations suggest that while AI-guided design represents a promising advance, substantial validation is required before clinical deployment.

### 5.3 Phage-CRISPR synergy and cocktails

To prevent phage-resistant mutants, recent studies have developed phage-CRISPR cocktails: multiple phages each delivering a different CRISPR system targeting distinct resistance genes or essential chromosomal genes (Fu et al., 2013). The mechanistic rationale for cocktail strategies is based on three principles: (i) targeting multiple essential functions reduces the probability of simultaneous escape mutations; (ii) combining lytic phage activity with CRISPR-mediated killing provides complementary mechanisms; and (iii) different phages can infect through distinct receptors, reducing the impact of receptor mutations.

For example, a three-phage cocktail delivering Cas9 (targeting *bla*KPC), Cas13 (targeting *ermB*), and a lytic phage (targeting the *lamB* receptor) eradicated a mixed resistant population of *K. pneumoniae* and *E. faecalis* in a murine wound infection model, with no regrowth after 14 days (Onyeyili et al., 2025). Clinical feasibility assessment of phage-CRISPR cocktails includes considerations of manufacturing complexity (multiple phages require separate production and quality control), regulatory pathways (combination product designation), and dosing regimens (optimal ratios and timing). Current evidence supports clinical feasibility for topical and localized infections, while systemic applications face additional challenges including immunogenicity and biodistribution (Fu et al., 2013).

Resistance frequencies to phage-CRISPR cocktails have been estimated at 10⁻⁸ to 10⁻¹⁰ per cell generation, compared to 10⁻⁶ to 10⁻⁷ for single phages and 10⁻³ to 10⁻⁶ for conventional antibiotics (Fu et al., 2013). This represents a substantial improvement, though practical emergence in clinical settings with large bacterial populations (10¹⁰−10¹² CFU) remains a concern Table 3, Table 4.

**Table 3:** Selected in vivo studies of CRISPR-Cas antimicrobials.

Animal model	Pathogen	Resistance gene	Delivery system	Sample size (n)	Statistical method	Control group	Treatment duration	Outcome (reduction in resistant CFU)	Ref.
Mouse skin infection	MRSA	*mecA*	Phagemid	n = 10/group	Student's *t*-test	Untreated, empty phage	7 days	4-log reduction, 80% survival	(Kiro et al., 2014)
Murine pneumonia	*K. pneumoniae*	*bla*KPC-2	Inducible CRISPRi + meropenem	n = 8/group	ANOVA with post-hoc	Untreated, meropenem alone	5 days	3-log reduction, restored sensitivity	(Meng et al., 2025)
Mouse thigh infection	*E. coli* ST131	*bla*CTX-M-15	Conjugative plasmid	n = 6/group	Mann-Whitney U	Untreated, empty plasmid	3 days	99.99% plasmid clearance	(Kiga et al., 2020)
Wound infection (mixed)	*K. pneumoniae* + *E. faecalis*	*bla*KPC + *ermB*	Phage cocktail	n = 12/group	Log-rank test	Untreated, single phage	14 days	No regrowth at day 14	(Fu et al., 2013)
Zebrafish sepsis	*P. aeruginosa*	*mexB* (efflux)	PLGA nanoparticles	n = 20/group	Kaplan-Meier	Untreated, empty nanoparticles	48 h	5-log reduction, 100% survival	(Wan et al., 2021)

**Table 4:** Translational challenges and proposed solutions.

Challenge	Description	Proposed solution	Ref.
Off-target cleavage	Cleavage of commensal genes	High-fidelity Cas variants (eSpCas9, HF1); AI-optimized guide design	(Saffari Natanzi et al., 2025; Noble et al., 2017)
Escape mutants	Mutations in target site or PAM	Multiplexed sgRNAs, essential gene targeting, relaxed PAM Cas variants	(Zetsche et al., 2015; Rottinghaus et al., 2022)
Horizontal CRISPR spread	Uncontrolled gene drive	Fitness cost + toxin-antitoxin kill switch, environmental containment	(Rottinghaus et al., 2022, Dagur)
Phage resistance	Bacterial receptor mutation	Phage cocktails + CRISPR targeting essential genes, receptor-independent phages	(Oechslin, 2018; Fu et al., 2013)
Immunogenicity	Pre-existing anti-Cas antibodies	Cas protein engineering, alternative orthologs, transient immunosuppression	(Noble et al., 2017)
Regulatory approval	No approved products	Standardized safety models, combination product pathway, phased clinical trials	(Pires et al., 2016)
Manufacturing	Low yield, high endotoxin	Continuous flow production, cell-free systems, endotoxin removal	(McGaw and Chong, 2021; Gootenberg et al., 2017)

## 6 Addressing translational challenges

### 6.1 Off-target effects and genotoxicity

CRISPR-Cas systems can cleave sequences with up to 5 mismatches, potentially targeting essential genes of commensal bacteria or the host microbiome (Onyeyili et al., 2025). A recent study using metagenomic sequencing of fecal samples treated with a Cas9-phage targeting *bla*CTX-M-15 revealed that 0.1% of off-target sites in commensal *Bacteroides* species were cleaved, leading to transient dysbiosis (Kleinstiver et al., 2016). To mitigate this, high-fidelity Cas9 variants (e.g., eSpCas9, SpCas9-HF1) have been engineered and shown to reduce off-target cleavage by >1000-fold while maintaining on-target activity (Rottinghaus et al., 2022).

### 6.2 Immunogenicity of Cas proteins and delivery vehicles

Immunogenicity represents a major translational barrier for CRISPR-Cas antimicrobials. Pre-existing antibodies against Cas9 derived from *S. aureus* (SaCas9) and *S. pyogenes* (SpCas9) are prevalent in human populations, with seropositivity rates estimated at 30–60% depending on the population and the specific Cas ortholog (Noble et al., 2017). These antibodies can neutralize Cas9 activity and trigger adverse immune responses upon administration. Additionally, phage capsid proteins elicit both innate and adaptive immune responses, including neutralizing antibodies that can limit efficacy and prevent repeat dosing.

Strategies to mitigate immunogenicity include: (i) engineering Cas9 variants with reduced immunogenicity while maintaining activity; (ii) developing Cas orthologs from less common bacterial species with lower seroprevalence; (iii) using transient immunosuppression regimens (e.g., corticosteroids, rapamycin) during administration; (iv) employing lipid nanoparticle delivery that reduces immunogenic exposure compared to phage-based systems; (v) PEGylation of phage capsids to reduce antibody recognition; and (vi) developing Cas9 with modified T-cell epitopes. Each strategy has advantages and limitations, and optimal approaches may require combination strategies tailored to specific clinical contexts. Clinical implications for repeat dosing are particularly significant, as many AMR infections require multiple treatment courses.

### 6.3 Horizontal gene transfer of CRISPR cassettes

A theoretical risk is that the CRISPR-encoding cassette could itself be transferred to unintended bacteria via conjugation or transduction, creating a "self-propagating" gene drive that might disrupt essential genes in environmental microbes (Rottinghaus et al., 2022). Mathematical modeling suggests that a fitness cost of >20% for carrying the CRISPR system prevents uncontrolled spread (Rottinghaus et al., 2022). Moreover, including a "kill switch" (e.g., a toxin gene under the control of a rare-sugar promoter) ensures that cells losing the CRISPR plasmid die, preventing horizontal escape (Dagur).

### 6.4 Bacterial resistance to CRISPR-Cas antimicrobials

Bacteria can evolve resistance to CRISPR-Cas antimicrobials through multiple mechanisms, which require detailed consideration for clinical translation:

(i) Anti-CRISPR (Acr) proteins: Over 100 Acr proteins have been identified across diverse bacterial species, with activities against Cas9, Cas12, Cas13, and other effectors. Acrs function through diverse mechanisms including direct binding to Cas proteins, inhibition of guide RNA loading, interference with target recognition, and blocking catalytic activity. The prevalence of Acrs in clinical bacterial populations is increasingly recognized, with some studies reporting Acr genes in 10–30% of clinical isolates, particularly in phage-exposed environments. Acr activity can substantially reduce therapeutic efficacy, and strategies to evade Acrs include engineering Acr-insensitive Cas variants, using Acr-targeting approaches, and developing Acr-resistant orthologs.
(ii) PAM mutations: Mutations in the protospacer-adjacent motif (PAM) can prevent Cas binding while preserving gene function. PAM mutation frequencies vary by target and bacterial species but have been observed at rates of 10⁻⁷ to 10⁻⁹ per generation. Strategies to overcome PAM mutations include using Cas variants with relaxed PAM requirements (e.g., SpCas9-NG, xCas9) and multiplexing sgRNAs targeting different PAM regions.
(iii) Spacer escape mutants: Deletions, insertions, or point mutations within the spacer-targeted region can prevent guide RNA recognition. These escape mutants are particularly problematic when targeting non-essential genes or when single sgRNAs are used. Multiplexing sgRNAs targeting multiple regions of the same gene or different genes reduces escape mutant frequency.
(iv) Phage resistance mechanisms: As discussed in Section 3.1, phage-resistant mutants emerge through receptor mutation, restriction-modification systems, CRISPR-Cas immunity in target bacteria, and superinfection exclusion. Phage-CRISPR cocktails and targeting of essential genes represent strategies to overcome these mechanisms.
(v) Fitness costs and compensatory evolution: CRISPR-Cas systems impose fitness costs on target bacteria, and compensatory mutations can restore fitness while maintaining resistance. Mathematical modeling suggests that fitness costs >20% prevent long-term persistence of resistance (Rottinghaus et al., 2022).

Clinical monitoring for resistance emergence will require rapid diagnostic approaches capable of detecting Acr genes, PAM mutations, and spacer escape mutants in patient samples. Integrating diagnostic and therapeutic CRISPR (theragnostic platforms) could enable real-time resistance monitoring and adaptive treatment strategies (Agha et al., 2025).

### 6.5 Regulatory and manufacturing hurdles

No CRISPR-based antimicrobial has yet received regulatory approval for human use. The U.S. FDA has designated several phage-CRISPR products as "combination products" (biologic + device), requiring extensive safety and toxicology studies (Pires et al., 2016). Manufacturing challenges include large-scale production of clinical-grade phages with consistent CRISPR payloads and removal of endotoxins (McGaw and Chong, 2021). However, recent advances in continuous-flow phage production and cell-free CRISPR ribonucleoprotein assembly have reduced production costs by 90% (Gootenberg et al., 2017).

## 7 Ecological and environmental considerations

### 7.1 Environmental reservoirs of AMR

Wastewater treatment plants, agricultural runoff, and natural water bodies are recognized hotspots for AMR dissemination. Environmental reservoirs harbor diverse bacterial communities where horizontal gene transfer occurs at high frequency, facilitating the spread of resistance genes. The application of CRISPR-Cas antimicrobials in clinical settings has the potential to release CRISPR constructs into these environmental reservoirs through patient excretion, wastewater discharge, and agricultural runoff. A recent study applied a Cas13-encoding phage cocktail directly to hospital wastewater, reducing the abundance of *bla*NDM, *mcr-1*, and *tet*(X) genes by >99% within 6 h without affecting total bacterial load (Sadeqi et al., 2022). However, the long-term ecological consequences of such interventions remain uncharacterized.

### 7.2 Risks of horizontal CRISPR dissemination

The potential for CRISPR systems to act as "gene drives" in natural bacterial populations represents a serious ecological concern. Mathematical modeling of CRISPR gene drive dynamics in bacterial populations suggests three key determinants of spread potential: (i) fitness cost of the CRISPR system (>20% prevents spread); (ii) transmission efficiency (conjugation or transduction rates); and (iii) population structure (spatial separation reduces spread). Current evidence suggests that well-designed CRISPR systems with fitness costs exceeding 20% are unlikely to spread uncontrollably in natural populations (Rottinghaus et al., 2022).

Ecological consequences of unintended CRISPR spread could include: (i) disruption of beneficial microbial communities; (ii) elimination of essential functions in environmental bacteria; (iii) horizontal transfer of CRISPR constructs to unintended species; (iv) co-selection of antibiotic resistance genes linked to CRISPR constructs; and (v) unintended evolutionary consequences for microbial ecosystems.

Containment strategies include: (i) genetic kill switches (toxin-antitoxin systems, temperature-sensitive elements); (ii) environmental containment (physical barriers, inactivation of CRISPR constructs before release); (iii) use of non-conjugative delivery platforms (nanoparticles, non-mobilizable phages); (iv) inclusion of CRISPR arrays targeting the CRISPR construct itself (self-targeting for containment); and (v) regulated expression systems that are active only in the presence of specific inducers.

Regulatory frameworks for environmental release of CRISPR constructs are currently under development, with the U.S. EPA and European Environmental Agency establishing guidelines for genetically modified organisms that include CRISPR-based systems. These frameworks emphasize risk assessment, environmental monitoring, and post-release containment measures. For clinical applications, regulatory agencies are developing specific guidelines for CRISPR therapeutics that include environmental risk assessment components (Pires et al., 2016).

### 7.3 Critical assessment of current evidence

A critical evaluation of the current evidence base for CRISPR-Cas antimicrobials reveals several important limitations. First, the gap between in vitro and in vivo results is substantial, with many promising strategies failing to translate to animal models due to issues of delivery, stability, and immune clearance. Second, current animal models (mouse, rat, zebrafish) have significant limitations for predicting human efficacy, including differences in immune responses, microbiome composition, and infection dynamics. Third, most in vivo studies have small sample sizes (n = 6–12 per group) and lack blinding or randomization, increasing the risk of bias. Fourth, the duration of follow-up in animal studies is typically short (<14 days), providing limited information on long-term safety, resistance emergence, or microbiome recovery. Fifth, there is a potential for confirmation bias in the literature, with positive results more likely to be published than negative or equivocal findings.

These limitations underscore the need for: (i) larger, well-powered animal studies with standardized protocols; (ii) long-term follow-up studies assessing safety, efficacy, and resistance emergence; (iii) randomized, blinded, multicenter animal studies to reduce bias; (iv) development of more predictive animal models (e.g., humanized mice, non-human primates); and (v) independent replication of key findings by multiple laboratories. Clinical translation should be approached with appropriate caution, with phase I human trials focusing on safety, pharmacokinetics, and pharmacodynamics before efficacy is evaluated in phase II/III trials.

### 7.4 Clinical translation: pharmacokinetics and pharmacodynamics

Pharmacokinetic (PK) and pharmacodynamic (PD) considerations are critical for the clinical development of CRISPR-Cas antimicrobials:

Biodistribution: Delivery platforms exhibit distinct biodistribution profiles. Phage-based systems are primarily retained in the bloodstream and liver, with limited tissue penetration. Nanoparticle systems can be engineered for tissue-specific delivery but face challenges in crossing biological barriers (blood-brain barrier, epithelial barriers). Conjugative plasmid systems distribute throughout the gastrointestinal tract but have limited systemic distribution.

Clearance and half-life: Phage clearance occurs primarily through the reticuloendothelial system with half-lives ranging from minutes to hours, depending on phage engineering and PEGylation. Nanoparticle clearance is determined by size, surface charge, and PEGylation, with half-lives ranging from hours to days. The short half-life of CRISPR components may limit efficacy for chronic infections but reduces immunogenicity risk.

Target site penetration: Penetration into infected tissues (lungs, skin, wounds) varies by delivery platform. Nanoparticles with surface targeting ligands can enhance tissue penetration, while phage penetration is limited in complex tissues with tight junctions. Biofilm penetration is particularly challenging, with EPS acting as a significant barrier for both phages and nanoparticles.

Dose-response relationships: The relationship between administered dose and antibacterial effect is complex and depends on delivery efficiency, bacterial susceptibility, and resistance emergence. Mathematical modeling suggests that achieving a 4-log reduction in bacterial load requires delivering CRISPR components to >99% of target cells, which is rarely achieved with current platforms.

PK/PD modeling: Integration of PK and PD parameters through mathematical modeling can guide dosing regimen optimization, predict resistance emergence, and identify optimal combination strategies with conventional antibiotics. Such models are still in early development for CRISPR therapeutics.

Implications for clinical development: PK/PD considerations suggest that: (i) multiple dosing may be required for efficacy; (ii) combination with antibiotics may be synergistic; (iii) administration routes (topical, intravenous, inhaled) must be optimized for each infection type; and (iv) patient-specific factors (immune status, microbiome composition) may influence PK/PD profiles.

## 8 Comparative analysis of CRISPR-Cas antimicrobials with other approaches

### 8.1 Systematic comparison

CRISPR-Cas antimicrobials must be evaluated in the context of other emerging approaches to combat AMR. Table 5 provides a systematic comparison across key parameters:

**Table 5:** Comparison of antimicrobial approaches for AMR.

Parameter	CRISPR-Cas	Conventional antibiotics	Phage therapy alone	Antimicrobial peptides	Probiotics	Monoclonal antibodies	Novel antibiotics
**Specificity**	High (sequence-specific)	Low (broad-spectrum)	Medium (species-specific)	Medium	Low	High (species/antigen-specific)	Variable
**Resistance development**	Low (multiplex targeting)	High	Medium	Low-Medium	Low	Low	Variable
**Mechanism of action**	Gene silencing/cleavage	Multiple (cell wall, protein synthesis, etc.)	Lytic killing	Membrane disruption	Competitive exclusion	Immune neutralization	Multiple
**Delivery challenges**	High (intracellular)	Low	High (phage-host range)	Medium	Low (oral)	Low (IV)	Low
**Immunogenicity**	Medium-High	Low	Medium	Low-Medium	Low	Medium	Low
**Spectrum of activity**	Narrow (target-specific)	Broad	Narrow	Broad	Narrow	Narrow	Variable
**Off-target effects**	Medium	High	Low	Low-Medium	Low	Low	Low-Medium
**Regulatory path**	Unclear (combination product)	Established	Emerging	Established	Established	Established	Established
**Manufacturing**	Complex	Established	Complex	Established	Established	Complex	Established
**Cost**	High (current)	Low	Medium	Low-Medium	Low	High	High
**Clinical readiness**	Preclinical/Phase I	Clinical	Phase I/II	Clinical	Clinical	Clinical	Clinical
**TRL**	3–5	9	5–7	7–8	8–9	7–9	6–9

CRISPR-Cas systems offer unique advantages in specificity, multiplex targeting, and potential to reverse resistance rather than simply killing bacteria. However, they face significant challenges in delivery, immunogenicity, and regulatory approval compared to other approaches. The optimal approach will likely be combination therapy, with CRISPR-Cas used to eliminate resistance genes while conventional antibiotics or phage provide initial bacterial killing.

### 8.2 Clinical development roadmap

**A concrete roadmap for clinical development of CRISPR-Cas antimicrobials is proposed:**

**Near-term (1–3 years):**

-Completion of ongoing phase I safety trials for topical phage-CRISPR products.

-Development of standardized immunogenicity assays and monitoring protocols.

-Establishment of Good Manufacturing Practice (GMP) guidelines for phage and nanoparticle production.

-Completion of preclinical toxicology studies in larger animal models.

-Regulatory engagement for phase II/III trial design.

**Mid-term (3–5 years):**

-Phase II efficacy trials for phage-CRISPR in chronic wound infections, cystic fibrosis-related pulmonary infections, and catheter-associated urinary tract infections.

-Development of predictive biomarkers for patient stratification.

-Establishment of dosing regimens and combination therapy protocols.

-Completion of environmental risk assessment studies.

-Regulatory approval for first phage-CRISPR combination product.

**Long-term (5–10 years):**

-Phase III trials for systemic infections.

-Development of theragnostic platforms for real-time resistance monitoring.

-Environmental deployment for wastewater treatment.

-Integration of AI-optimized guide design into clinical workflows.

-Establishment of global regulatory framework for CRISPR therapeutics.

-Cost-effectiveness analysis and healthcare implementation studies.

**Critical milestones for success include:**

-Demonstration of safety in >500 human subjects.

-Consistent manufacturing with <10% batch-to-batch variability.

-Achievement of >4-log reduction in bacterial load in target tissues.

-Prevention of resistance emergence in >95% of treated patients.

-Favorable cost-effectiveness compared to conventional therapy for resistant infections.

## 9 Future directions and diagnostic integration

### 9.1 Theragnostic platforms

CRISPR-Cas systems are not only therapeutic but also serve as rapid diagnostics for AMR. The DETECTR and SHERLOCK platforms can detect resistance genes directly from clinical specimens (blood, urine, sputum) in under 1 h with attomolar sensitivity (Yosef et al., 2015). Integrating diagnostic and therapeutic CRISPR into a single "theragnostic" platform is an emerging concept: a portable device would first identify the resistance profile of an infection, then synthesize and deliver the appropriate sgRNA and Cas effector on-site (Agha et al., 2025). Proof-of-concept has been demonstrated for carbapenem-resistant Enterobacteriaceae using a lyophilized all-in-one reaction mix (Agha et al., 2025).

### 9.2 Environmental applications

Another frontier is the use of CRISPR to disrupt resistance genes in environmental reservoirs. Wastewater treatment plants are hotspots for AMR dissemination. A recent study applied a Cas13-encoding phage cocktail directly to hospital wastewater, reducing the abundance of *bla*NDM, *mcr-1*, and *tet*(X) genes by >99% within 6 h without affecting total bacterial load (Naghavi et al., 2024; Sadeqi et al., 2022). However, this study was limited to laboratory-scale reactors, and translation to full-scale wastewater treatment plants will require careful consideration of environmental factors, regulatory approval, and ecological monitoring.

### 9.3 Future research priorities

Based on the current state of the field, the following research priorities are identified:

(i) Cas protein engineering: Development of Cas variants with reduced immunogenicity, enhanced specificity, relaxed PAM requirements, and resistance to Acr proteins. High-fidelity Cas9 variants (eSpCas9, SpCas9-HF1) represent progress, but further engineering is needed for clinical applications. Cas orthologs from less common bacterial species with lower seroprevalence should be characterized.
(ii) Delivery platform optimization: Improvement of delivery efficiency to Gram-positive bacteria, biofilm penetration, and systemic administration. Novel biomaterials (hydrogels, sustained-release formulations) and combination delivery strategies (e.g., phage-nanoparticle hybrids) should be explored.
(iii) Resistance mitigation strategies: Development of predictive models for resistance emergence, rapid diagnostics for Acr genes and escape mutants, and algorithms for dynamically updating CRISPR guides. Phage-CRISPR cocktails targeting essential genes and multiplexed sgRNA designs represent promising approaches.
(iv) Combination therapies: Systematic evaluation of CRISPR-Cas combinations with conventional antibiotics, phage, antimicrobial peptides, and immune modulators. Synergy screening approaches and mathematical modeling can guide optimal combination strategies.
(v) Diagnostic integration: Development of portable, rapid diagnostic platforms that can identify resistance genes and guide CRISPR therapy selection. Integration with artificial intelligence for real-time data analysis and treatment optimization.
(vi) Clinical trial design: Development of standardized protocols for phase I, II, and III trials, including safety monitoring, immunogenicity assessment, and efficacy endpoints. Regulatory engagement and standardization of manufacturing processes.
(vii) Environmental applications: Assessment of ecological risks and benefits, development of containment strategies, and establishment of regulatory frameworks for environmental deployment. Monitoring of long-term effects on microbial communities and resistance gene reservoirs.
(viii) Cost-effectiveness analysis: Comprehensive economic evaluation of CRISPR-Cas antimicrobials compared to conventional therapy, including costs of manufacturing, delivery, monitoring, and healthcare savings from reduced AMR burden.

## 10 Conclusion

CRISPR-Cas systems have evolved from a bacterial curiosity to a versatile platform for precision antimicrobial development. Unlike conventional antibiotics that exert broad-spectrum killing and accelerate resistance, CRISPR-based approaches can be programmed to selectively eliminate specific resistance genes, cure plasmids, or transiently silence resistance mechanisms. The past three years have witnessed remarkable advances: novel Cas effectors (Cas3, Cas13, Cas14), diverse delivery platforms (OMVs, nanoparticles, bacterial ghosts), intelligent guide design using AI, and sophisticated systems like ATTACK that prevent horizontal escape. In vivo efficacy has been demonstrated in multiple animal models, and the first human trials for phage-CRISPR products are anticipated by 2027.

However, significant hurdles remain. Immunogenicity of Cas proteins and phage capsids represents a major translational barrier, with pre-existing antibodies in 30–60% of the population. Off-target effects in commensal bacteria and the host microbiome require careful evaluation and mitigation. Bacterial resistance mechanisms including anti-CRISPR proteins, PAM mutations, and spacer escape mutants must be addressed through multiplex targeting and engineering approaches. Ecological risks of horizontal CRISPR dissemination and environmental impacts require comprehensive assessment and containment strategies. Regulatory pathways remain undefined, manufacturing scalability is unproven, and cost-effectiveness has not been demonstrated.

The most promising delivery platform for near-term clinical translation appears to be phage-CRISPR for topical or localized infections (chronic wounds, cystic fibrosis-related pulmonary infections), where delivery is less challenging and immunogenicity is more manageable. Nanoparticle systems may become more feasible for systemic applications with further optimization of targeting and reduced immunogenicity. Conjugative plasmid systems show promise for gastrointestinal applications but require kill switches to prevent horizontal spread.

**Fig. 3:**
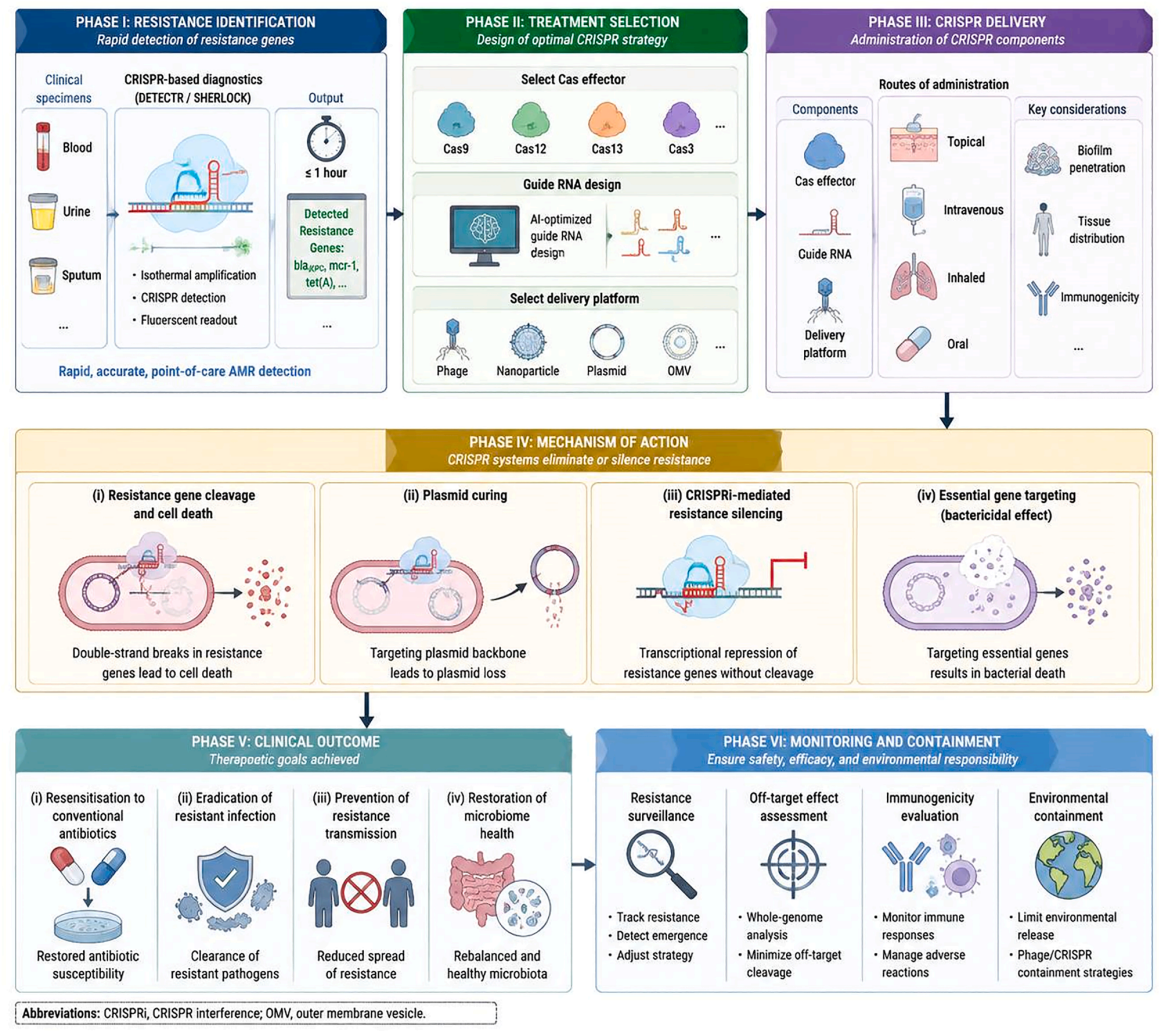
**Comprehensive workflow for CRISPR-Cas antimicrobial therapy.** Integrated schematic depicting the complete pathway from resistance identification through treatment to clinical outcome. **Phase I: Resistance identification.** Clinical specimens (blood, urine, sputum) are analyzed using CRISPR-based diagnostics (DETECTR, SHERLOCK) to detect resistance genes within 1 h. **Phase II: Treatment selection.** Based on the identified resistance profile, optimal Cas effector (Cas9, Cas12, Cas13, Cas3), guide RNA design (AI-optimized), and delivery platform (phage, nanoparticle, plasmid, OMV) are selected. **Phase III: CRISPR delivery.** Components are administered via the appropriate route (topical, intravenous, inhaled, oral) with consideration of biofilm penetration, tissue distribution, and immunogenicity. **Phase IV: Mechanism of action.** Delivered CRISPR systems achieve: (i) resistance gene cleavage and cell death; (ii) plasmid curing; (iii) CRISPRi-mediated resistance silencing; or (iv) essential gene targeting for bactericidal effect. **Phase V: Clinical outcome.** Therapeutic goals include: (i) resensitization to conventional antibiotics; (ii) eradication of resistant infection; (iii) prevention of resistance transmission; and (iv) restoration of microbiome health. **Phase VI: Monitoring and containment.** Post-treatment monitoring includes resistance surveillance, off-target effect assessment, immunogenicity evaluation, and environmental containment measures. Abbreviations: CRISPRi, CRISPR interference; OMV, outer membrane vesicle.

The path to clinical implementation requires: (i) completion of well-powered, randomized, blinded animal studies with long-term follow-up; (ii) development of standardized manufacturing and quality control protocols; (iii) establishment of regulatory frameworks for phage-CRISPR combination products; (iv) phase I human trials focusing on safety, immunogenicity, and PK/PD; (v) development of rapid diagnostic tools for patient selection and resistance monitoring; and (vi) comprehensive environmental risk assessment. We have created a comprehensive schematic (Fig. 3) that integrates all elements.

The integration of CRISPR diagnostics and therapeutics into a single theragnostic workflow promises to transform the management of resistant infections from a reactive to a predictive and personalized paradigm. As we stand at the precipice of a post-antibiotic era, CRISPR-Cas systems offer not just an alternative but a fundamentally smarter way to combat antimicrobial resistance—provided that the scientific, regulatory, and ecological challenges are addressed with appropriate rigor and caution.

## GenBank accession number

No new nucleotide sequences were generated or deposited; therefore, a GenBank accession number is not applicable.

## Funding

No specific funding was received for this work.

## AI declaration

No artificial intelligence (AI) tools were used in the preparation of this manuscript.

## Ethical approval

Ethical approval was not required for this study as it is a literature review and involved no human or animal subjects, nor any primary experimental data from patient samples.

## CRediT authorship contribution statement

**Mahsa Khosrojerdi:** Writing – original draft, Resources, Formal analysis, Data curation. **Seyed Ahmad Hashemi:** Writing – original draft, Software, Methodology, Data curation. **Reza Besharati:** Writing – review & editing, Software, Methodology, Data curation. **Ali Haghbin:** Writing – original draft, Methodology, Investigation, Conceptualization. **Amir Azimian:** Writing – review & editing, Writing – original draft, Supervision, Software, Resources, Project administration, Methodology, Investigation, Formal analysis, Data curation, Conceptualization.

## Declaration of competing interest

The authors declare that they have no known competing financial interests or personal relationships that could have appeared to influence the work reported in this paper.

## Data Availability

No data was used for the research described in the article.
